# ABC Cardiol — O Lar da Pesquisa Científica Cardiovascular

**DOI:** 10.36660/abc.20201206

**Published:** 2020-12-01

**Authors:** Carlos E. Rochitte

**Affiliations:** 1 Universidade de São Paulo Faculdade de Medicina Hospital das Clínicas São PauloSP Brasil Universidade de São Paulo Faculdade de Medicina Hospital das Clínicas Instituto do Coração, São Paulo, SP - Brasil; 2 Hospital do Coração São PauloSP Brasil Hospital do Coração (HCOR), São Paulo, SP – Brasil

**Keywords:** Bibliometria, Métricas, Publicações Periódicas, Fator de Impacto de Revistas, Revisão da Pesquisa por Pares

Os Arquivos Brasileiros de Cardiologia, ou ABC Cardiol, revista oficial da Sociedade Brasileira de Cardiologia, constituem o mais importante periódico científico de divulgação de publicações científicas cardiovasculares no Brasil. Em 2018, como o novo editor-chefe, propus duas abordagens principais para aumentar ainda mais a relevância do ABC Cardiol na comunidade científica. A primeira foi aumentar o fator de impacto do nosso periódico, que havia se estabilizado ligeiramente acima de 1, e a segunda foi aumentar a internacionalização do periódico, conforme recomendado pelo Scielo.^1^

**Figura 1:**
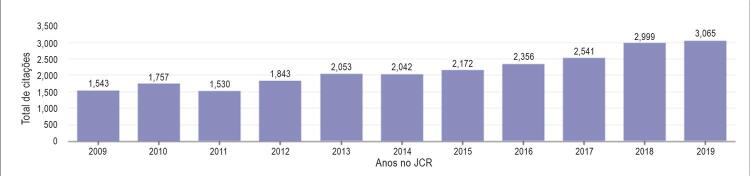
– Número total de citações do ABC Cardiol por ano (Journal of Citation Reports — JCR — Clarivate).

**Figura 2:**
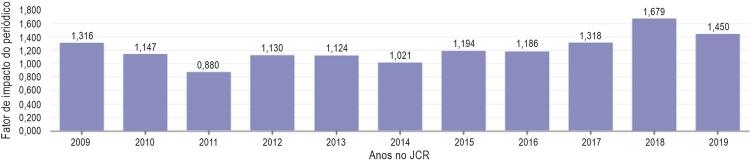
– Fator de Impacto de 2 anos do ABC Cardiol, de 2009 a 2019 (Journal of Citation Reports — JCR — Clarivate).

**Figura 3:**
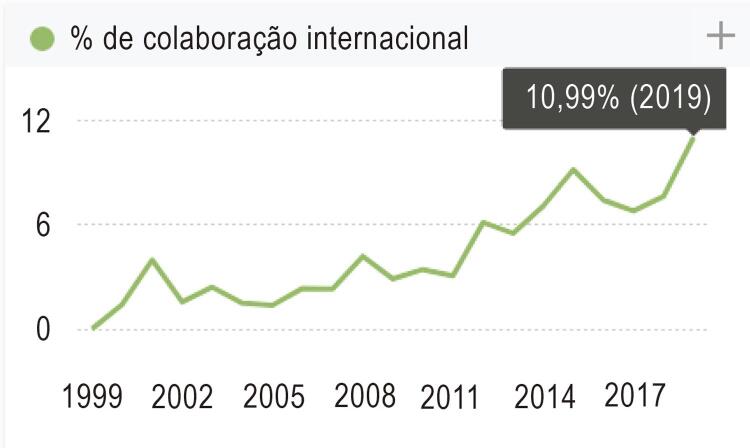
– Número de artigos do ABC Cardiol assinados por mais de um país por ano, de 1999 a 2019 (Scimago Journal).

**Figura 4:**
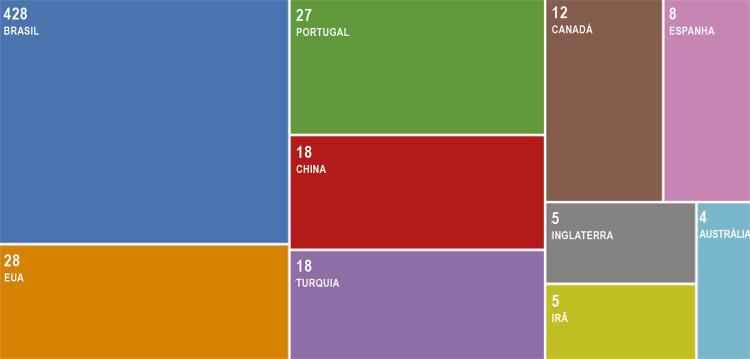
– Número de publicações do ABC Cardiol por país em 2018 e 2019 (Web of Science — Clarivate).

Nosso fator de impacto mais recente (2019) é 1.450 e o fator de impacto de 5 anos é 1.724, o mais alto de nossa história. Além disso, o número de citações em um determinado ano também atingiu o maior número de todos os tempos: 3.065 citações em apenas um ano ( Figura 1 ). Houve uma tendência de aumento do fator de impacto do nosso periódico, passando de 1.318 (2017) e o impacto mais alto em 1.679 (2018)^2 , 3^ ( Figura 2 ). A colaboração internacional tem aumentado significativamente. Atualmente, cerca de 11% dos nossos artigos publicados são de autores de mais de um país (multinacionais, Figura 3 ), e 22% dos artigos são originários de outros países que não o Brasil ( Figura 4 ). Nosso conselho editorial tem agora editores associados internacionais que são a pedra angular para aumentar a colaboração internacional e as publicações no ABC Cardiol. Além disso, a colaboração com a comunidade científica internacional e as sociedades se intensificou.^4^

Também aumentamos nossos requisitos de excelência para artigos e revisões.^5^ As diretrizes e declarações se tornaram uma fonte educacional de atualização de conhecimentos para profissionais da área médica. O ABC Cardiol implementou uma linha contínua de publicações científicas de diretrizes de alta qualidade que vão desde prevenção e estilo de vida até a cardiologia avançada. Esse papel específico tem um vínculo intrínseco com nossa responsabilidade social como periódico científico que é levar os benefícios da ciência à nossa sociedade.

Apesar desse desenvolvimento significativo no ABC Cardiol, ainda lutamos para obter o reconhecimento de um de nossos ranques de periódicos nacionais (QUALIS) fornecido pela agência nacional para programas de pós-graduação, CAPES (Coordenação de Aperfeiçoamento de Pessoal de Nível Superior).^6^ A tentativa correta da instituição de ter apenas uma nota de classificação para cada periódico científico, independentemente de sua área ou categoria específica, tropeçou na utilização de percentis de cada área de pesquisa. Por exemplo, o fator de impacto mais alto e o número de periódicos com fator de impacto muito alto variam consideravelmente de acordo com áreas de pesquisa específicas. Isso torna as comparações de percentis entre periódicos pertencentes a diferentes subáreas de pesquisa completamente imprecisas e injustas. Na categoria de sistema cardíaco e cardiovascular que inclui a Cardiologia, onde o ABC Cardiol está inserido, o maior fator de impacto é 23,603 com muitos periódicos com fatores de impacto extremamente altos. Isso constitui um uso indevido do fator de impacto dos periódicos.^7 , 8^ Nenhum dos fatores como os elevados padrões éticos, apoio de nossa sociedade profissional, publicação simultânea em inglês e no idioma local, nosso uso intenso das mídias sociais e nossa rede de estudiosos e acadêmicos jamais foram considerados pela CAPES. Isso representa uma falta de compreensão do que é ciência relevante, que deve incluir o verdadeiro impacto dos artigos acadêmicos, não apenas uma novidade, mas também a adequação dos métodos, a solidez ética e a relevância para a comunidade científica local e global. O QUALIS CAPES foi pego em muitas falácias como o argumento dedutivo, indutivo, de autoridade, ad hominem.^7^ Correções urgentes são necessárias antes que danos graves sejam causados de maneira definitiva à Ciência Brasileira.

Lidar com números frios de pontuações de classificação para periódicos é um negócio complexo e enganoso.^9 , 10^ No entanto, uma abordagem mais cívica e patriótica, com o objetivo final de encaminhar a ciência e os cientistas brasileiros por meio do uso de periódicos científicos nacionais como veículo para divulgar sua melhor ciência é o caminho certo a seguir.

Por fim, apesar da injustiça de nossas instituições nacionais, continuaremos buscando o objetivo de manter o ABC Cardiol como uma publicação científica de excelência em Cardiologia, e consolidar a ciência em nossa sociedade.
