# Supplementary material for: ABC Cardiol — O Lar da Pesquisa Científica Cardiovascular
Source: Arq Bras Cardiol. 2020 Dec 1;115(6):1044–6. [Article in Portuguese] doi: 10.36660/abc.20201206 (PMC8133717; doi:10.36660/abc.20201206)
Supplement: Translation

# Translation: ABC Cardiol – Our Home of the Cardiovascular Scientific Research

**Authors:** Carlos E. Rochitte

**Affiliations:** 1 Brazil Universidade de São Paulo Faculdade de Medicina Hospital das Clínicas Instituto do Coração, São Paulo, SP - Brazil; 2 Brazil Hospital do Coração (HCOR), São Paulo, SP – Brazil

**Keywords:** Bibliometrics, Metrics, Periodicals, Journal Impact Factor, Peer Review, Research

ABC Cardiol, the Arquivos Brasileiros de Cardiologia, an official journal of the Brazilian Society of Cardiology has been the premier scientific journal for conveying cardiovascular science publications in Brazil. In 2018 as the new editor-in-chief, I proposed two main approaches to further increase ABC Cardiol relevance in the scientific community. The first one was to increase our journal impact factor, which had been stabilized slightly above 1 and, secondly to increase the internationalization of the journal, which was recommended by Scielo.^1^

Our most recent journal impact factor (2019) is 1.450 and the 5-year impact factor of 1.724, the highest in our history. Additionally, the number of citations in a given year also reached its all-time highest number of 3065 citations in just one year ( Figure 1 ). There has been an upward trend of our journal impact factor, from the previous 1.318 (2017) and highest impact at 1.679 (2018)^2 , 3^( Figure 2 ). International collaboration has been increasing significantly. Currently, about 11% of our published articles are from authors of more than one country (multinational, Figure 3 ), and 22% of articles are originating from countries other than Brazil ( Figure 4 ). Our editorial board has now international associated editors that are the angular stone for increasing international collaboration and publications in the ABC Cardiol. Moreover, collaboration with the international scientific community and societies has been boosted.^4^

**Figure 1:** – ABC Cardiol number of total citations per year (Journal of Citation Reports – JCR – Clarivate).

**Figure 2:** – ABC Cardiol 2-year Journal Impact Factor from 2009 to 2019 (Journal of Citation Reports – JCR – Clarivate).

**Figure 3:** – ABC Cardiol number of articles signed by more than one Country per year, from 1999 to 2019 (Scimago Journal).

**Figure 4:** – ABC Cardiol number of publications by Country in 2018 and 2019 (Web of Science – Clarivate).

We have also increased our requirements for excellent articles and reviews.^5^Guidelines and statements have become an educational source of updating knowledge for medical professionals. ABC Cardiol has implemented a continuous line of scientific publications of high-quality guidelines ranging from prevention, and lifestyle to advanced cardiology. This specific role has an intrinsic link to our social responsibility as a scientific journal that is to bring the benefits of science to our society.

Despite this significant development in the ABC Cardiol, we still struggle to get recognition from one of our national journal rank (QUALIS) provided by our country agency for post-graduation programs, CAPES (Coordenação de Aperfeiçoamento de Pessoal de Nível Superior).^6^The agency correct attempt to have only one rank score for each scientific journal, independently of its specific area or category stumbled by the use of percentiles of each research area. For example, the highest impact factor and the number of very high impact factor journals vary considerably according to the specific research area. This makes percentile comparisons between journals belonging to different research sub-areas completely inaccurate and unfair. In the cardiac and cardiovascular system category that includes Cardiology, where ABC Cardiol is inserted, the highest impact factor is 23.603 with many journals with extremely high impact factors. This constitutes a clear misuse of Journal impact factor.^7 , 8^None of the critical factors such as high ethical standards, support of our professional society, publication in English and local language simultaneously, our very intense use of social media, and our scholar and academic network were ever considered by CAPES. This represents a lack of understanding of what is relevant science, which should include the true impact of academic articles, not only a novelty but also methods adequacy, ethical soundness, and relevance to the scientific community locally and globally. The QUALIS from CAPES was captured in many fallacies such as the deductive, inductive, argument from authority, and ad hominem argument.^7^Urgent corrections are needed before severe damage is definitively made to Brazilian Science.

Dealing with cold numbers of Journals rank scores is a tricky business,^9 , 10^nonetheless, a more civic and patriotic approach, with the ultimate goal of forwarding Brazilian science and scientists through using national scientific journals as the vehicle for communicating their best science is the right way to go.

Finally, despite the unfairness of our national agencies, we will continue to pursue the goal of maintaining ABC Cardiol as a scientific publication of excellence in Cardiology and consolidate science in our society.
